# Computational Study of the Inhibition of RgpB Gingipain, a Promising Target for the Treatment of Alzheimer’s Disease

**DOI:** 10.1021/acs.jcim.2c01198

**Published:** 2023-01-17

**Authors:** Santiago Movilla, Sergio Martí, Maite Roca, Vicent Moliner

**Affiliations:** BioComp Group, Institute of Advanced Materials (INAM), Universitat Jaume I, 12071 Castellón, Spain

## Abstract

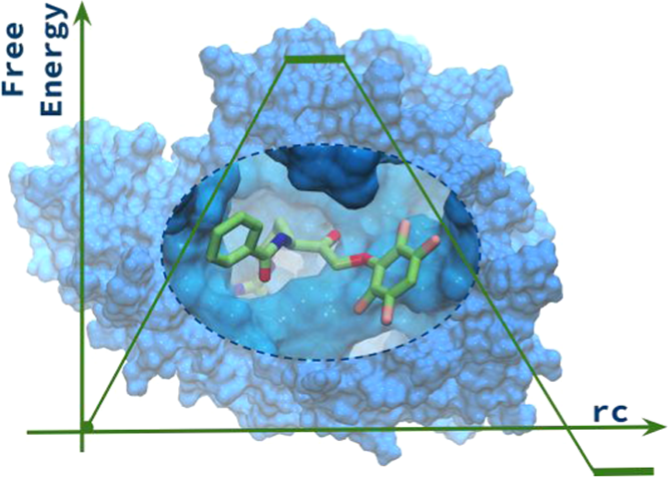

Alzheimer’s disease represents one of the most ambitious challenges for biomedical sciences due to the growing number of cases worldwide in the elderly population and the lack of efficient treatments. One of the recent attempts to develop a treatment points to the cysteine protease RgpB as a promising drug target. In this attempt, several small-molecule covalent inhibitors of this enzyme have been proposed. Here, we report a computational study at the atomic level of the inhibition mechanism of the most promising reported compounds. Molecular dynamics simulations were performed on six of them, and their binding energies in the active site of the protein were computed. Contact maps and interaction energies were decomposed by residues to disclose those key interactions with the enzyme. Finally, quantum mechanics/molecular mechanics (QM/MM) molecular dynamics (MD) simulations were performed to evaluate the reaction mechanism by which these drug candidates lead to covalently bound complexes, inhibiting the RgpB protease. The results provide a guide for future re-design of prospective and efficient inhibitors for the treatment of Alzheimer’s disease.

## 1 Introduction

Gingipains are a group of enzymes secreted by the pathogenic bacterium *Porphyromonas gingivalis*.^1^ Although their activity is mainly related to oropharyngeal problems, there are reports that also associate them with other health disorders.^2−5^ Some of these disorders are of particular medical interest, such as heart conditions and Alzheimer’s disease.^2,6−11^ The increasing number of neurodegenerative diseases worldwide and the lack of an appropriate medical treatment explains why this is the greatest current challenge for biomedical sciences.

Gingipains are a small family of cysteine protease enzymes which catalyze the cleavage of peptidic bonds in several protein substrates.^12^ In this family, we can find two types of gingipains, Lys-gingipains (Kgp), and Arg-gingipains (Rgp), classified according to the residue that they recognize at the P1 position, lysine and arginine, respectively, to perform the cleavage.^12^ Without extra marked preferences for other amino acids at positions beyond P1, gingipains catalyze the proteolysis of a large number of proteins and peptides.^13,14^ This makes them potentially harmful to the host integrity. Indeed, a recent study strongly linked one of the Rgp, RgpB, to the progression of Alzheimer’s disease.^6^ This study also showed that RgpB inhibition resulted in a strong arrest of disease progression in mice. With this study, RgpB was positioned as an important pharmacological target to understand its function and selectively inhibit it.

The proteolysis reaction mechanism of RgpB was recently studied by computational methods and reported in detail by our group.^15^ In this study, we highlight several critical features to understand the catalytic mechanism of this cysteine protease and proceed to its inhibition. In general, and as is common in cysteine proteases, the mechanism is divided into an acylation step and a deacylation step.^16−26^ According to our results,^15^ the deprotonation of the nucleophilic cysteine of RgpB in the acylation stage is performed by the substrate itself in response to the steric impossibility of the catalytic histidine to perform this process. Similar mechanisms for activation have been reported in enzymes whose active site distribution coincides with that of RgpB,^18^ where the substrate is positioned between the Cys/His catalytic dyad. After the deprotonation of the catalytic Cys, the sulfur atom of Cys attacks the carbonyl group of the substrate to obtain the acylenzyme. The deacylation stage is carried out in a single step where a water molecule attacks the carbonyl group of the substrate and one proton of the water is transferred to the sulfur atom of the Cys residue assisted by the catalytic His.

The interest in the inhibition of gingipains has been growing since their potential implications in the treatment of neurodegenerative diseases have been demonstrated.^27^ In fact, some potential drug candidates, which have been patented (International Patent Application PCT/US2015/054050 and PCT/US2016/061197), are in advanced stages of medical trials. Interestingly, these compounds were reported to be irreversible inhibitors after the corresponding kinetic tests had been carried out.^6^ Given the pharmacological potential of these molecules, the main goal of the present study is to investigate how these irreversible inhibitors can stop the catalytic activity of RgpB as a protease.

Structurally, these inhibitors have the side chain pattern of arginine but without the nitrogen of the P1–P1′ peptide bond. The absence of this nitrogen makes it impossible to carry out the hydrolysis reaction, breaking the peptide bond, thus inhibiting the enzyme. Interestingly, they also lack the reactive warheads commonly used for the covalent inhibition of cysteine proteases.^20,28−30^ This fact limits the reactive possibilities of these compounds against the action of RgpB gingipain. In our previously reported study,^15^ it was proposed that, in the absence of a peptidic nitrogen, the activation of catalytic cysteine was carried out by the proton transfer from the sulfur atom of Cys to the oxygen atom of the carbonyl group of the substrate.^15^ Based on these results, we proposed that this mechanism could be exploited for the design of potential irreversible drugs.

This background and in view of the limited reactive nature of the already irreversible patented inhibitors, a detailed understanding of their inhibition mechanism is necessary to exploit their potential in the future. Namely, in the case of covalent inhibitors, the inhibitory potency derives from the synergy between the noncovalent interactions with the enzyme and the kinetics/thermodynamics of the reactive process.^29^ Therefore, an atomic-level study of the inhibition mechanism must incorporate both the study of the inhibitor–enzyme interactions and the study of the covalent binding reaction mechanism. Here, we present a comprehensive computational study that provides insights into important atomic details in the reaction mechanism of inhibition, binding affinity, and the interaction between the enzyme and a representative group of inhibitors. Classical molecular dynamics (MD) simulations, alchemical transformations,^31^ molecular mechanics Poisson–Boltzmann surface area (MM/PBSA)^32^ calculations supplemented with interaction entropies, and MD simulations with quantum mechanics molecular mechanics (QM/MM) potentials were carried out to get a detailed description of the inhibitor–enzyme binding step and the chemical steps of the covalent inhibitor–enzyme bond formation. The obtained results throw light on the design of better inhibitors for Alzheimer’s disease treatment.

## 2 Methods

### 2.1 System Set Up

**Scheme 1:**
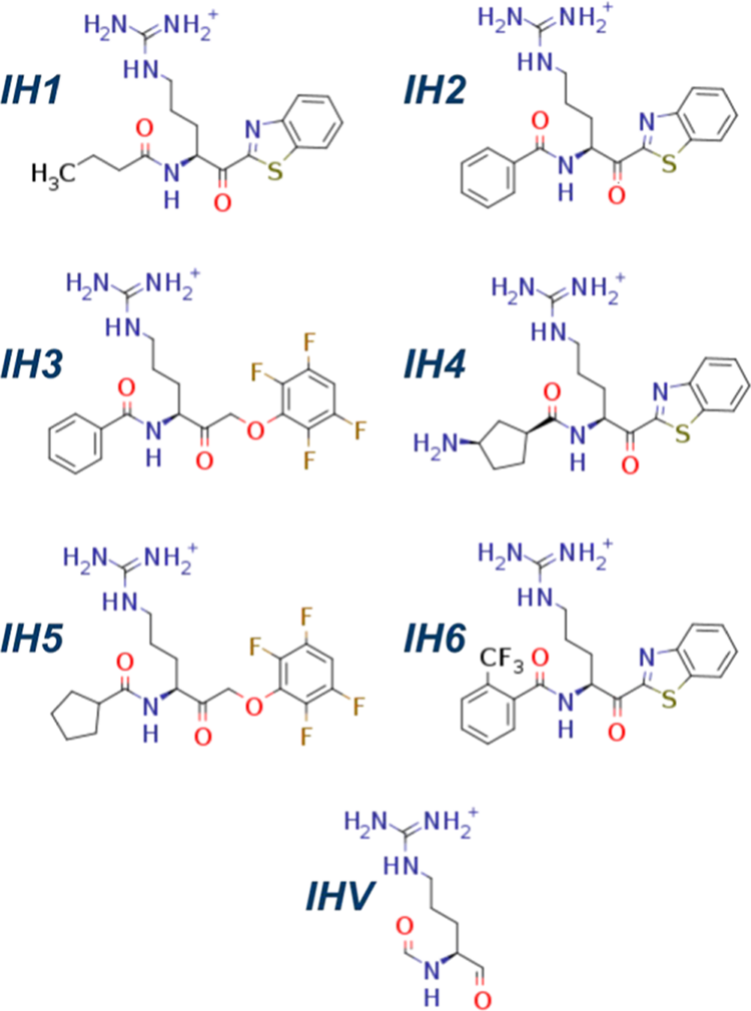
Chemical Structures of the Studied Irreversible Inhibitors of RgpB (IH1–IH6) and Sketch of the Virtual Inhibitor (IHV) Used as the Common Point for the Alchemical Transformations

The initial coordinates of the system were obtained from the previously equilibrated model^15^ of RgpB in complex with a short peptide. This model was reported in the reactivity study of RgpB performed by our group. The peptide was replaced by six active inhibitors, (Scheme 1) reported in International Patent Application PCT/US2016/061197, which covered the largest extent of the chemical space. The protonation state of all titratable residues was verified using the PROPKA3 server^33^ at reference pH 7.5,^6,13,14^ no atypical protonation states were observed. Each of the systems was solvated with a cubic box of TIP3P water^34^ molecules with a minimum distance of 15 Å between any solute atom and the edge of the box. A total number of 18 Na^+^ ions were added until the systems were neutralized.

### 2.2 Classical Molecular Dynamics (MD)

Protein and water molecules were described using the AMBERff14SB^35^ and TIP3P^34^ force field parameters, respectively. Monovalent ions were treated using the parameters proposed by Joung–Cheatham.^36^ For inhibitors, restrained electrostatic potential (RESP) charges^37^ were obtained from HF/6-31G* calculations on optimized geometries with the B3LYP functional^38,39^ with the 6-31G* basis set, according to previously reported protocols.^40^ The remaining force field parameters for the inhibitors were assigned from the small-molecules generalized Amber force field (GAFF).^40^ The topologies of each system were obtained using the tLEaP package from Ambertools20.^41^

All MD classical simulations were carried out in several steps: (i) solvent molecules, ions, and hydrogens were minimized using 2500 steps with the conjugate gradient algorithm; (ii) 200 ps of MD simulations of the solvent molecules and monovalent ions were carried out, with the positions of the backbone atoms restrained; (iii) two energy minimizations, one with the protein backbone restrained and another fully unrestrained were done for each system; (iv) the whole systems were heated gradually in three consecutive NPT simulations from 100 to 310 K, with a constant pressure of 1 bar; and finally, (v) 250 ns of NPT MD simulations were performed at 310 K and 1 bar. All parameters of the classical simulations were replicated from the previously reported study.^15^ A 10 Å cutoff was set up for short-range nonbonded interactions while a Particle Mesh Ewald (PME)^42,43^ model was used for the long-range interactions. Langevin dynamics thermostat^44,45^ was applied with a 3 ps^–1^ collision frequency. For all equilibration simulations, the SHAKE algorithm^46,47^ was used to constrain light atoms, and the velocity Verlet algorithm^48^ was used to propagate the system.

### 2.3 MM/PBSA and Interaction Entropies

To estimate the binding free energy (Δ*G*_bind_) of the inhibitors, calculations of MM/PBSA^32^ supplemented with corrections to the solute entropy using the methodology proposed by Duan et. al.^49^ were performed. For these calculations, frames were taken every 500 ps from each classical MD simulation. MM/PBSA calculations were performed with an implicit salt concentration of 150 mM (to reproduce experimental conditions)^6,13^ and using MMPBSA.py as implemented in Amber20.^41^ To obtain the binding free energy (Δ*G*_bind_), we applied the interaction entropy approach^49^ to compute the entropic contribution term (−*T*Δ*S*) at 310 K.

### 2.4 Alchemical Calculations

To obtain accurate relative values of the binding free energy (ΔΔ*G*_bind_), we performed alchemical transformations^31^ from the inhibitors to a virtual ligand (IHV) connecting them all (Scheme 1). For this purpose, the 3-step Amber Thermodynamic Integration protocol (“decharge–LJ–recharge” protocol)^50^ was used. Each transformation was carried out in ten windows equally distributed throughout the λ range (0–1). In each window, 500 ps of CPU equilibration (pmemd.MPI) and 5 ns of sampling with GPU algorithms (pmemd.cuda) were performed.^51,52^ Only the atoms appearing/disappearing during the transformation were included in the soft-core region. All the simulations were performed at the NVT ensemble at 310 K, starting from the volume-equilibrated structures of the classical MD trajectories. For data analysis, the first 5% of the simulation time (250 ps) of each window was discarded.

### 2.5 Potential of the Mean Force (PMF)

**Scheme 2:**
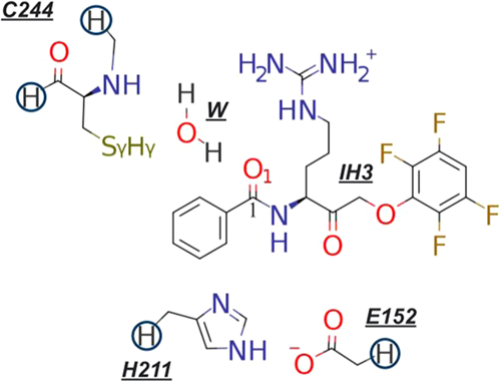
Schematic Representation of the Region Treated Quantum Mechanically to Explore the Inhibitory Mechanism of Binding between IH3 and RgpB The link atoms are shown as hydrogens inside circles. The water molecule (W) was included in the QM region when exploring the mechanism with the participation of a water molecule.

The six studied inhibitors present the same reactive carbonyl warhead (C1:IH3–O1:IH3, Scheme 2). Then, the inhibitor that showed the most favorable binding energy was selected for studying the reaction mechanism of the formation of the inhibitor–enzyme covalent complex by generating the free energy surfaces (FES) in terms of potential of mean force (PMFs). For the PMF calculations, two-dimensional (2D) potential energy surfaces (PES) were first computed through sequential minimizations along selected collective variables (CV) that best describe the reactions. A conjugate gradient algorithm was employed for the minimizations using a gradient tolerance of 0.1 kcal mol^–1^ as a convergence criterion. Later, the corresponding FES were generated at 310 K taking the structures obtained along the PES as starting points. Each window had a relaxation time of 5 ps and a sampling time of 25 ps using a time step of 0.5 fs in the NVT ensemble. Temperature control was performed using Langevin dynamics with a 3 ps^–1^ collision frequency.^44,45^ The umbrella sampling method^53^ was used to restrain the reaction coordinates. The force constant used for each window was 580 kcal mol^–1^ Å^–2^, and the window width was 0.05 Å for those collective variables corresponding to the antisymmetric combination of two distances and 0.1 Å for those distances. The number and the width of the windows selected ensure a correct overlapping of windows. The umbrella integration method, implemented in the QM3 suite,^54^ was used to reweight the biased sampling dynamics and to generate the PMFs along selected coordinates. The quantum mechanical (QM) region used for the generation of the PMFs is shown in Scheme 2. The method used to describe the QM region was PM6.^55^ Following the same protocol of our previous study on this system,^15^ a structure close to the transition state region was selected and the transition state structure was located at the PM6/MM level and verified by tracing down the intrinsic reaction coordinate path (IRC). The obtained minima were optimized. Single point energy calculations were carried out on the stationary point structures at the PBE-D3(BJ)^56−60^/MM level with the 6-311+G** basis set, to correct the PM6/MM potential energy. The thermal contributions calculated by the statistical methods at the PM6/MM level were thus preserved. To verify the results, all the critical points were reoptimized at the PBE-D3(BJ)/MM level using the QM3 suite.^54^

## 3 Results and Discussion

### 3.1 Binding Affinities (Δ*G*_bind_) and Inhibitor–Enzyme Interaction Profiles

To study the noncovalent interaction profiles between inhibitors and RgpB enzyme, 250 ns of classical MD simulations were run per inhibitor in the inhibitor–enzyme noncovalent reactant complex. None of the systems, in the presence of the corresponding inhibitor, exhibited significant changes in the protein structure (all backbone RMSDs <2 Å on average, Figure S1).

Starting from the equilibrated structures of the classical MD simulations, we initially proceeded to estimate the binding free energies for each of the compounds. For this purpose, we computed the binding affinity energies by MM/PBSA^32^ calculations supplemented with a correction term to the entropic contributions based on the interaction entropies proposed by Duan et al.^49^ (Table 1). Qualitatively speaking, compounds IH3 and IH4 proved to produce the most stable inhibitor–enzyme complexes. It should also be noted that IH3 is the one that shows the most favorable binding energy in both entropic and enthalpic terms. On the other hand, inhibitor IH2 presented a lower binding enthalpy and a higher −*T*Δ*S* term. We emphasize that the values computed by means of MM/PBSA and interaction entropies have no quantitative meaning and are purely used to qualitatively analyze the enthalpic and entropic contributions of the binding processes.^61^ Thus, keeping in mind the inherent uncertainty associated with the MM/PBSA method, alchemical transformations^31^ were employed to compute the differences between the binding free energies of every inhibitor and the virtual inhibitor IHV (ΔΔ*G*_bind-TI_) to obtain more precise and quantitatively meaningful inhibitor–enzyme affinities.

**Table 1:** Binding Free Energies for the Studied Inhibitors Obtained over the Classical MD Simulations Using MM/PBSA and Interaction Entropies (Δ*G*_bind-MM/PBSA_) and Binding Free Energies Relative to the Virtual Inhibitor (IHV) Computed by Means of Alchemical Transformations (Δ*G*_bind-TI_)^a^

Inhibitor	Δ*H*_bind-MM/PBSA_	–*T*Δ*S*_bind-MM/PBSA_	Δ*G*_bind-MM/PBSA_	ΔΔ*G*_bind-TI_
IH1	–57.3	37.0	–20.3	0.20
IH2	–54.2	50.6	–3.6	5.42
IH3	–69.4	27.3	–42.1	0.55
IH4	–67.2	28.2	–39.0	1.99
IH5	–63.3	27.8	–35.5	2.55
IH6	–60.9	33.1	–27.8	3.31

a All values are in kcal mol^–1^.

The results of the alchemical transformations show a similar trend to the one obtained from MM/PBSA calculations, with the only exception of IH1, previously ranked as the second weakest, is repositioned in first place with practically the same ΔΔ*G*_bind-TI_ as IH3. The same as the MM/PBSA calculations,^32^ IH4 ranks after IH3 as one of the most potent candidates. Likewise, IH2 is the inhibitor with the lowest affinity to the enzyme with a difference of 5.2 kcal mol^–1^ with respect to IH1. Henceforth, IH3 can be considered as the reference inhibitor given the agreement between the binding free energies estimated by both methods.

**Figure 1:**
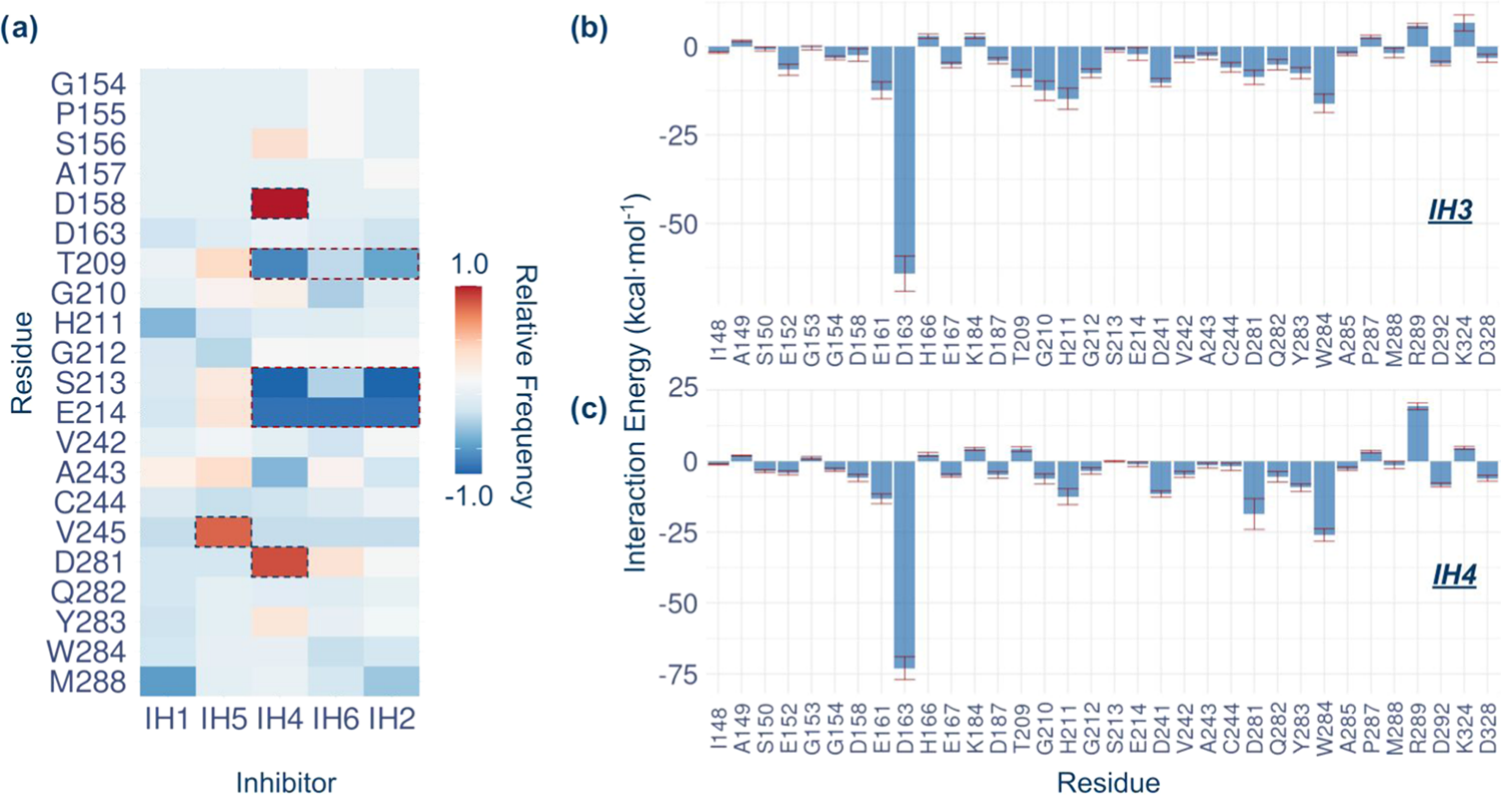
(a) Relative frequency of contacts between residues of RgpB and the inhibitors. IH3 was used as a reference and a contact was counted if the distance between atoms was <4.0 Å. The cells corresponding to the interactions that differ the most from the IH3 inhibitor are highlighted with dashed-line borders. Panels (b, c) show averaged interaction energies (electrostatic plus Lennard–Jones) between residues of RgpB and IH3 and IH4, respectively, over 1 ns MD simulations at the PM3/MM level.

**Figure 2:**
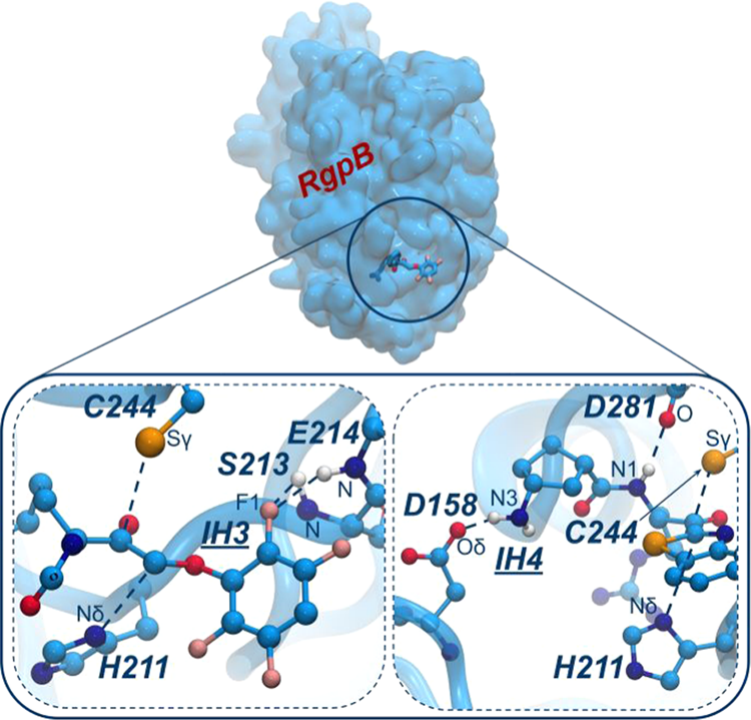
Top panel: surface representation of the whole protein and location of the binding pocket. IH3 inhibitor is represented as sticks. Bottom panels: Schematic representation of the key interactions between IH3 (left) or IH4 (right) and the binding pocket of RgpB.

A contact frequency map allowed us to analyze the differences in the interaction patterns/profiles of each inhibitor, which can be complemented with the analysis of the averaged inhibitor–enzyme interaction energies decomposed by the residue (Figure 1). Figure 1a shows the relative contact frequencies with respect to the inhibitor with the highest affinity, IH3. Thus, positive values represent a higher contact frequency than in IH3 while negative values represent a lower contact frequency than in IH3. In general, most of the inhibitors present a similar interaction profile with the enzyme, which is in agreement with the differences observed between the binding free energies (ΔΔ*G*_bind-TI_) computed by alchemical transformations. However, it can be observed from the contact map that three inhibitors (IH2, IH4, and IH6) show a significantly lower contact frequency with residues Ser213 and Glu214 (see Figure 1a). A visual inspection revealed that for the case of inhibitors IH3 and IH5 this interaction corresponds to a hydrogen bond between the HN:Glu214 and the F1 atom of the inhibitor. In the case of the interaction with the Ser213 residue does not form a hydrogen bond due to the angle of the atoms involved (angle around 59 ± 16°) (Figure 2). Thus, for those inhibitors that instead of the tetrafluorophenoxy substituent have the benzothiazole ring, the interactions with Ser213 and Glu214 are not present. However, IH1 manages to interact with HN:Glu214 via the nitrogen atom of the bicyclo ring. Although no preferences in selectivity of the groups around the arginine moiety have been reported in RgpB that affect the catalytic capacity, it has shown a higher affinity for hydrophobic substituents, mainly aromatic.^13^ However, these results suggest that hydrogen bonding groups are necessary to stabilize these aromatic substituents within the active site and can be optimized to achieve better affinity to the enzyme.

Another interaction worth highlighting is the one observed between compound IH4 and residues Asp158 and Asp281 (see Figures 1a and 2). Namely, IH4 is the only one of the compounds studied that presents a hydrogen bond donor at the nitrogen substituent group of the arginine. Consequently, HN3:IH4 manages to interact with Oδ:Asp158 (Figure 2). As a result, this inhibitor is repositioned in such a way that it manages to form an extra hydrogen bond interaction between the HN1:IH4 and O:Asp281 atoms. Although this interaction would be possible in all inhibitors, it was only observed in IH4, suggesting that it responds to the conformation adopted due to the interaction with the Asp158 residue. Other differences in the contact map, such as those observed in residues Thr209 or Val245, are less specific and are the consequence of a particular physical proximity during the simulations.

Finally, to energetically characterize the favorable observed contacts between the inhibitors and the RgpB residues, the interaction energies were calculated (Figures 1b,c and S3–S6). We can observe the determinative role played by residues Asp163, Trp284, and His211 in arginine binding, a conclusion that was predicted from structural analysis and our previous report on the proteolysis reaction catalyzed by RgpB.^15^ This result is not surprising given the high selectivity of RgpB to arginine residues. On the other hand, it can be observed that the interaction with residues Ser213 and Glu214 is always favorable. In the same way, the interaction between IH4 and residues Asp158 and Asp281 shows a lower energy, being remarkable for the one with Asp281. In contrast, residues Lys184, Arg289, and Lys324 show an unfavorable interaction energy with all compounds. Although this may be a starting point for future optimizations, it derives from the spatial proximity (without contact) between the charges of these residues and the positive charge of the guanidinium group of the arginine moiety of the inhibitors.

To sum up, our results suggest that the optimization should be focused on compounds presenting hydrogen bond acceptor groups capable of interacting with HN:Glu214 and HN:Ser213. Moreover, hydrogen bond donor groups interacting with Asp158 and Asp281 seem to enhance the affinity with the enzyme. On the other hand, the guanidinium group present in all inhibitors and in the natural substrate should be preserved as has been shown to be essential for recognition by the RgpB binding pocket.

### 3.2 Covalent Binding Chemical Step

To study the reaction mechanism by which these inhibitors covalently bind to the enzyme, QM/MM MD simulations were performed to generate the full free energy landscapes of the most plausible mechanisms. Given the similarity between the inhibitors, the reactivity study was carried out using only the IH3 inhibitor, which showed the most favorable binding energy.

**Figure 3:**
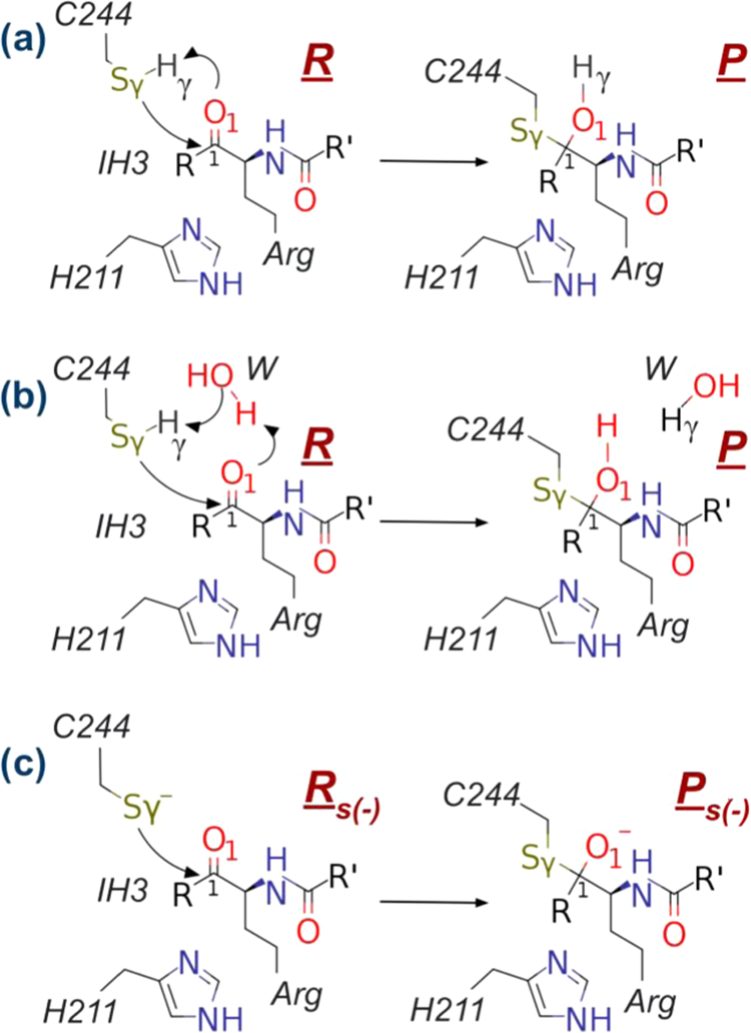
Schematic representation of the considered mechanisms of covalent binding between IH3 and RgpB. Sketch of the reaction mechanism in which transfer of the Hγ:Cys244 to the O1 atom of the inhibitor occurs (a) directly or (b) mediated by a water molecule. (c) Representation of the reaction mechanism starting from the deprotonated Sγ:C244 form. As shown in Scheme 1 for IH3, R and R′ correspond to phenyl and 2,3,5,6-tetrafluorophenoxy substituents, respectively.

**Figure 4:**
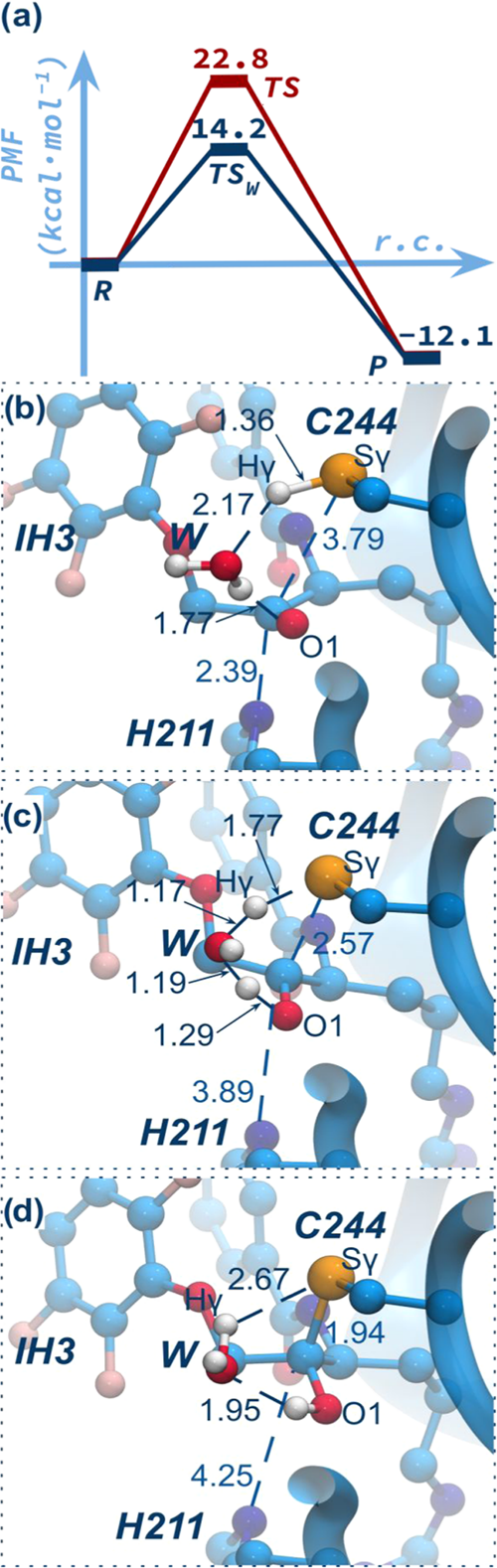
(a) Free energy profiles computed for the inhibitory covalent binding mechanisms of RgpB by IH3 at the PBE + D3(BJ):PM6/MM. The direct proton transfer mechanism from Cys244 to O1 atom of IH3 is depicted in red line while the proton transfer mechanism mediated by a water molecule is shown in blue line. PBE + D3(BJ)/MM optimized structures of the critical points (b) R, (c) TS_W_, and (d) P along the most likely covalent binding mechanism of IH3 inhibiting RgpB catalytic activity. Selected distances are in Å. FESs are shown in Figure S7.

Initially, and analogously to the proteolysis reaction catalyzed by the enzyme in the presence of wild-type substrate, Cys244 is protonated and requires activation to attack the carbonyl group in the reactant state (R in Figures 3a,b and 4b). We also considered the possibility that the mechanism proceeded from the deprotonated Sγ:Cys244 form (mechanism R_S(-)_ → P_S(-)_ in Figure 3c). However, several attempts (with and without restraints) to obtain stable reactive structures were unsuccessful. In all simulated cases, the negative charge on Cys244 resulted in deformations of the system to chemically unviable structures.

The family of inhibitors reported does not possess a clear reactive moiety, such as a Michael acceptor or an epoxide group. Instead, they only possess the carbonyl oxygen atom O1:IH3 as a possible acceptor/activator for the cysteine residue. This possibility was previously hypothesized^15^ in light of the small difference between the free energy barriers shown by the wild-type being activated by either the peptide nitrogen (more favorable mechanism by ∼1.1 kcal mol^–1^) or carbonyl oxygen of the substrate. Other possible bases were evaluated by calculating the population of hydrogen bonds between Hγ:Cys244 and other possible proton acceptors. However, the interaction with O1:IH3 was shown to populate more than 50% of the time, while other possible bases, such as Asp281, interacted less than 1% with it, making them poor base candidates. With the carbonyl group as the only plausible activator of Cys244, the mechanisms are limited to whether the attack of Sγ:Cys244 on C1:IH3 and the transfer of Hγ:Cys244 from Sγ:Cys244 to O1:IH3 occur directly or mediated by a water molecule (Figure 3a,b).

As revealed by the computed FESs (Figure S7), both mechanisms, the one in which the Hγ:Cys244 is directly transferred to the O1:IH3 atom (mechanism a in Figure 3) and the one mediated by a water molecule (mechanism b in Figure 3), proceed through a single concerted step. In the case of the direct mechanism, that was explored using the Sγ:Cys244–C1:IH3 distance and the difference between distances [Sγ:Cys244–Hγ:Cys244] – [O1:IH3–Hγ:Cys244] as collective variables to describe the process, the activation free energy associated with the transition state (TS, see Figure 4a) is 22.8 kcal mol^–1^ with respect to the reactants. A value that is very close to that previously computed for the natural substrate, 23.5 kcal mol^–1^.^15^^15^However, in the case of the transition state mediated by a water molecule (TS_W_), that was explored using the distance Sγ:Cys244–C1:IH3 and the difference between distances [Sγ:Cys244–Hγ:Cys244] – [O:W–Hγ:Cys244] as collective variables to describe the process, the corresponding activation energy is 14.2 kcal mol^–1^. This result suggests that this mechanism is the most viable for the covalent binding reaction with a significantly lower free energy barrier compared to that of the wild-type substrate. The obtained product (P) is located at −12.1 kcal mol^–1^ with respect to the reactants, thus giving an irreversible exergonic reaction. To verify the mechanism obtained at the PM6/MM level, R, TS_W_, and P were fully optimized at a higher level of theory, PBE + D3(BJ)/MM. The high-level optimized structures, presented in Figure 4, match the reaction pathway predicted at a lower level, verifying the mechanism deduced from PM6/MM FES.

As mentioned above, the mechanism starting from the deprotonated Cys244 (mechanism c in Figure 3) was not explored because the active site was deformed along the classical MD simulations and no appropriately reactive structures were obtained.

In light of these results, it is remarkable to highlight the ability of RgpB to react without the presence of highly reactive groups. Common reactive groups such as Michael acceptors or epoxides deal with selectivity issues due to their reactivity with other undesired targets. The unusual carbonyl warhead enables to exploit the design of covalent inhibitors, with high potency, without having to face problems of selectivity. These features qualify RgpB as a pharmacological target that promises effective treatments by the use of these kinds of inhibitors without side effects for the treatment of Alzheimer’s disease.

## 4 Conclusions

Alzheimer’s disease is one of the most studied medical challenges today. Gingipains proteases, including RgpB, have positioned themselves as potential drug targets for the development of treatments for the disease. Herein, we unveil atomic-level details of the noncovalent binding processes for a set of RgpB gingipain inhibitors, potential candidates for the treatment of Alzheimer’s disease. Six compounds, presenting the side chain pattern of arginine but without the nitrogen of the P1–P1′ peptide bond, were chosen to cover the widest breadth of the chemical space previously proposed and patented,^6^ some of them are in advanced stages of clinical testing. Initially, noncovalent inhibitor–enzyme complexes were simulated by classical molecular dynamics. Over the MD sampling, binding free energies (ΔΔ*G*_bind-TI_) were computed with alchemical transformation calculations.

Based on a relative contact map analysis between the inhibitors and the enzyme, we concluded that IH3, IH1, and IH5 present similar patterns of interactions, showing only minor differences between the contacts established with the protein residues along the sampled trajectories. Meanwhile, among IH2, IH4, and IH6, some of them rendered lower binding energies, and do not interact with key residues Ser213 and Glu214. However, IH4 exhibits two hydrogen bonding interactions that no other shows with Asp158 and Asp281 residues. These interactions as well as those with Ser213 and Glu214 were found to have a favorable interaction energy whenever present. The analyses of the noncovalent complexes suggest that IH1 and IH3 would be the most promising candidates for further refinements, according to their high binding affinities to the active site of RgpB.

The reaction mechanism of the covalent bond formation between the inhibitors and the enzyme was computed by QM/MM MD simulations on IH3, which showed the best binding profile in the noncovalent complex. The most plausible reaction mechanism proceeds through a single concerted step, in which activation/deprotonation of Cys244 is carried out by the O1:IH3 atom mediated by a water molecule (W). At the same time, the Sγ:Cys244 atom attacks the C1:IH3 of the inhibitor to give way to a stable product (−12.1 kcal mol^–1^). The reaction proceeds through an activation barrier (14.2 kcal mol^–1^) significantly lower than that reported previously, computed in our laboratory for the wild-type substrate (23.5 kcal mol^–1^).^15^ Thus, RgpB has a remarkable ability to react without the presence of highly reactive groups, enabling to exploit the re-design of covalent inhibitors, recognized for their potency, without predicting problems of selectivity. These results qualify RgpB as a pharmacological target that promises effective treatments by the use of these kinds of inhibitors without side effects. In particular, the inhibitors should contain a hydrogen bond donor and acceptor groups to be able to interact with Glu214, Ser213, Asp158, and Asp281, and conserving the reported guanidinium group and warhead to provide potency and selectivity. The interactions reported here and the mechanistic details represent a key starting point for future re-design of prospective and efficient inhibitors for the treatment of Alzheimer’s disease.

## Data Availability

Amber14 Package can be purchased from ambermd.org/GetAmber.php. QM3 Suite is freely available via a public GitHub repository github.com/sergio-marti/qm3. AmberTools17 can be obtained from ambermd.org/AmberTools.php. Gaussian 09 D01 can be purchased from gaussian.com. fDYNAMO v2.2 can be freely downloaded from www.pdynamo.org/downloads. Force field parameters for IH3 and IHV are reported in the Supporting Information together with a complete IH3 inhibitor/protein/ions/solvent box PDB file. The coordinates of the QM region for the critical points located at the PBE + D3(BJ)/MM level are also reported in the Supporting Information. Examples of the inputs for classical MD, alchemical transformations and MMPBSA calculations are openly available on the AMBER website.

## Supporting Information Available

Root-mean-square deviation (RMSD) analyses for classical MD simulations; root-mean-square fluctuation (RMSF) analyses for classical MD simulations; averaged interaction energies between the inhibitors and some protein residues for IH1, IH2, IH5, and IH6; free energy surfaces for the covalent binding inhibition mechanisms of the RgpB gingipain with IH3; force field parameters for IH3 and IHV; cartesian coordinates for R, TS_W_, and P structures localized at the PBE + D3(BJ)/MM level for the covalent binding mechanism with the IH3 inhibitor (PDF)
A full structure of the solvated protein in complex with the IH3 inhibitor (PDB)

## Supplementary Material

ci2c01198_si_001.pdf

ci2c01198_si_002.pdb
