# Supplementary material for: Computational Study of the Inhibition of RgpB Gingipain, a Promising Target for the Treatment of Alzheimer’s Disease
Source: J Chem Inf Model. 2023 Jan 17;63(3):950–8. doi: 10.1021/acs.jcim.2c01198 (PMC10882967; doi:10.1021/acs.jcim.2c01198)
Supplement: Supplementary file 1 — ci2c01198_si_001.pdf [file ci2c01198_si_001.pdf]

Supporting Information

Computational Study of the Inhibition of RgpB Gingipain, a Promising Target for the Treatment of Alzheimer's Disease

Santiago Movilla[1], Sergio Martí[1], Maite Roca[1,*] and Vicent Moliner[1,*]

[1]BioComp Group, Institute of Advanced Materials (INAM), Universitat Jaume I, 12071, Castellón, Spain

[*]Correspondence should be addressed to:

Maite Roca: mroca@uji.es

Vicent Moliner: moliner@uji.es

Table of Contents

Figure S1 ………………………………………………………………………… S3

Figure S2 ………………………………………………………………………… S4

Figure S3 ………………………………………………………………………… S5

Figure S4 ………………………………………………………………………… S6

Figure S5 ………………………………………………………………………… S7

Figure S6 ………………………………………………………………………… S8

Figure S7 ………………………………………………………………………… S9

Force Field Parameters ………………………………………………………… S10

Cartesian coordinates of the QM sub-set of atoms ……..………..……….. S26

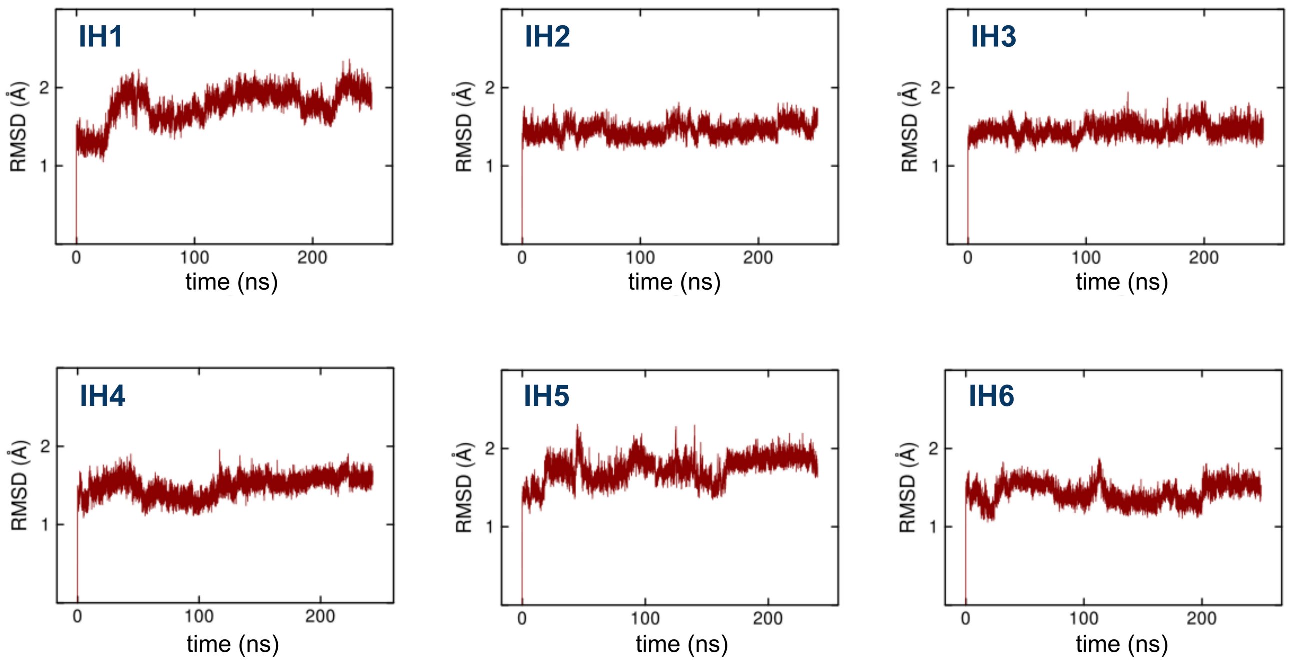

Figure S1. Time evolution of the root-mean-square deviation (RMSD) of protein backbone atoms (Cα, C, N, O) of the RgpB gingipain in complex with the six selected inhibitors (IH1 to IH6).

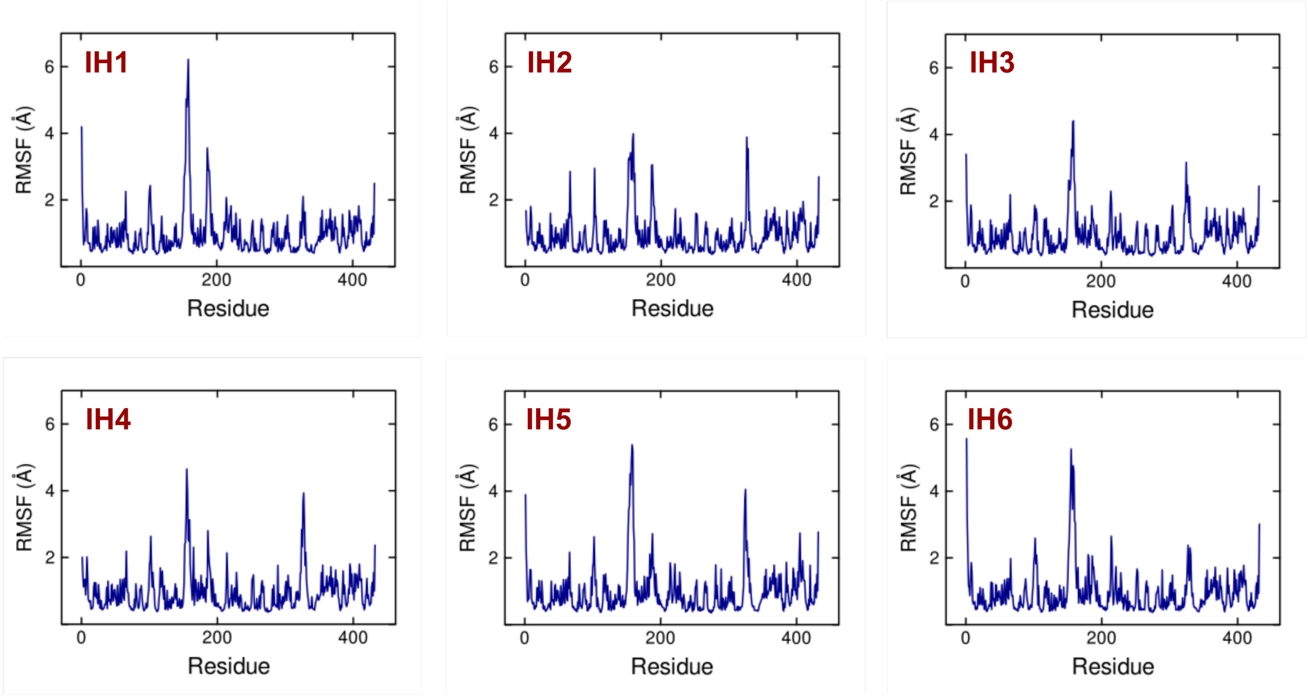

Figure S2. Root-mean-square fluctuation (RMSF) analysis of protein α carbon atoms of the RgpB gingipain in complex with the six selected inhibitors (IH1 to IH6).

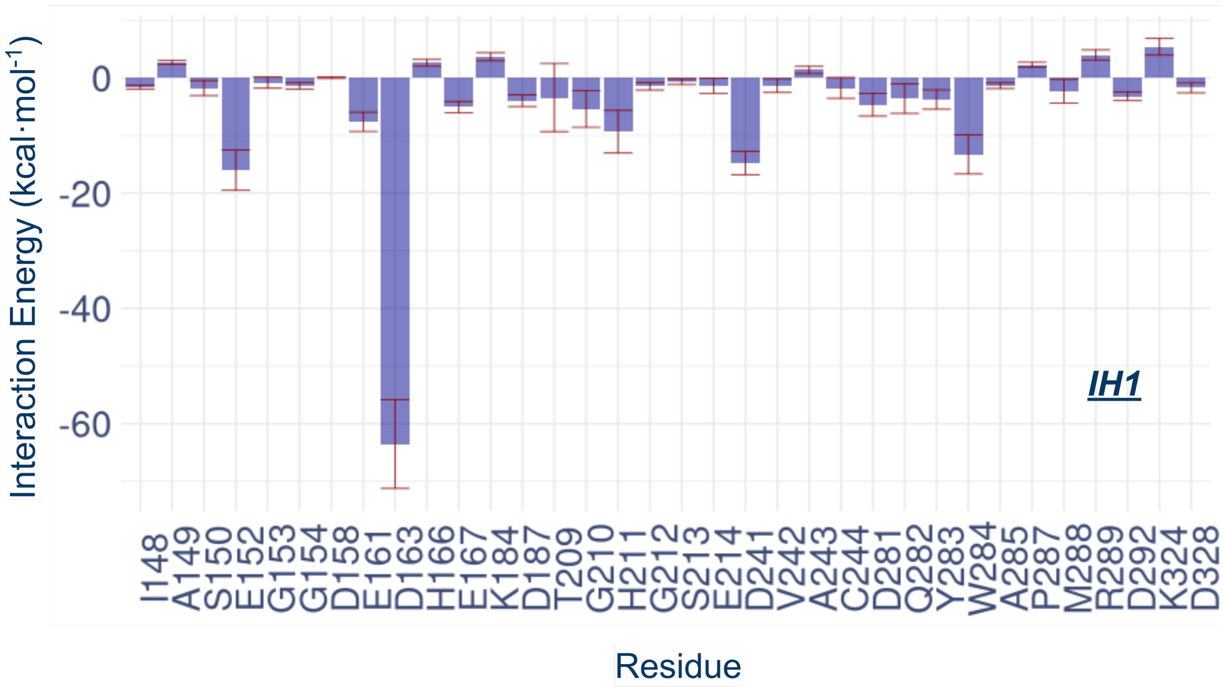

Figure S3. Averaged interaction energies (electrostatic plus Lennard−Jones) between some residues of RgpB and IH1.

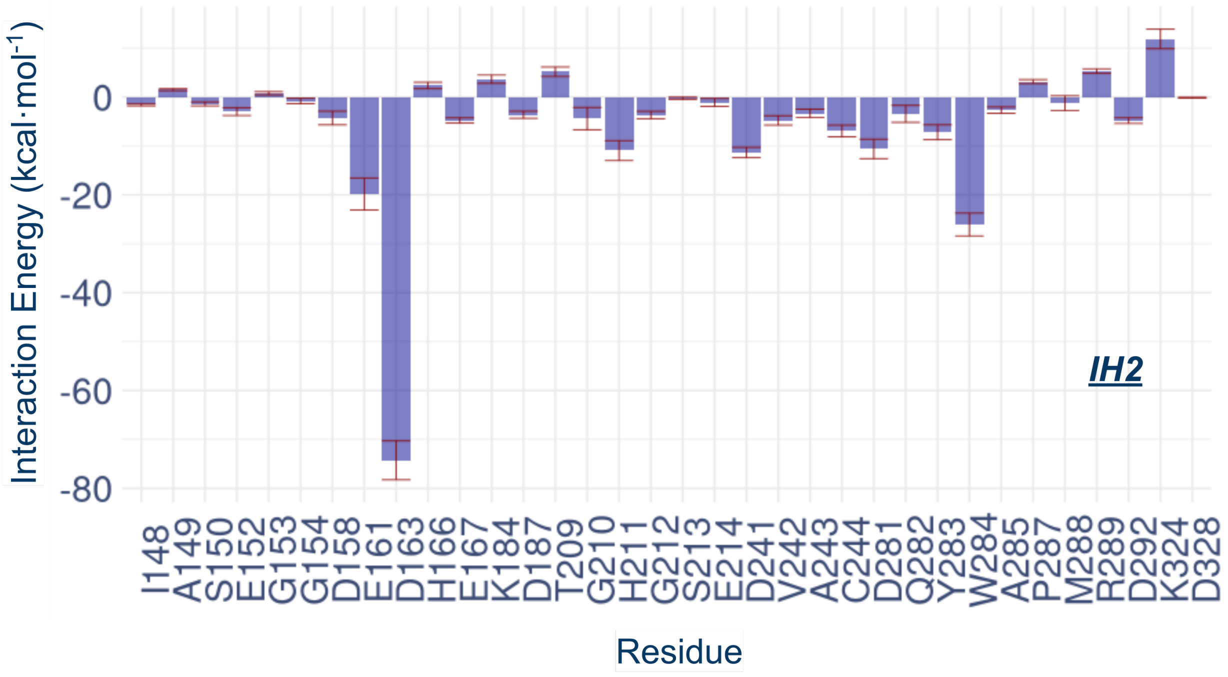

Figure S4. Averaged interaction energies (electrostatic plus Lennard−Jones) between some residues of RgpB and IH2.

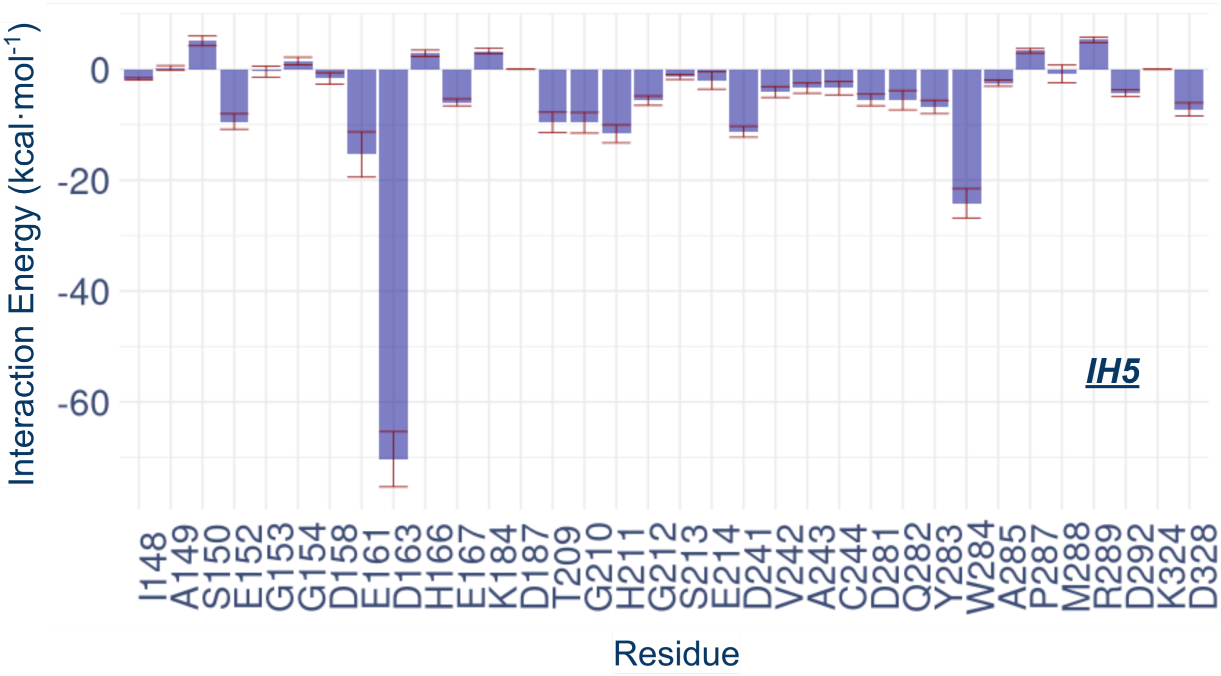

Figure S5. Averaged interaction energies (electrostatic plus Lennard−Jones) between some residues of RgpB and IH5.

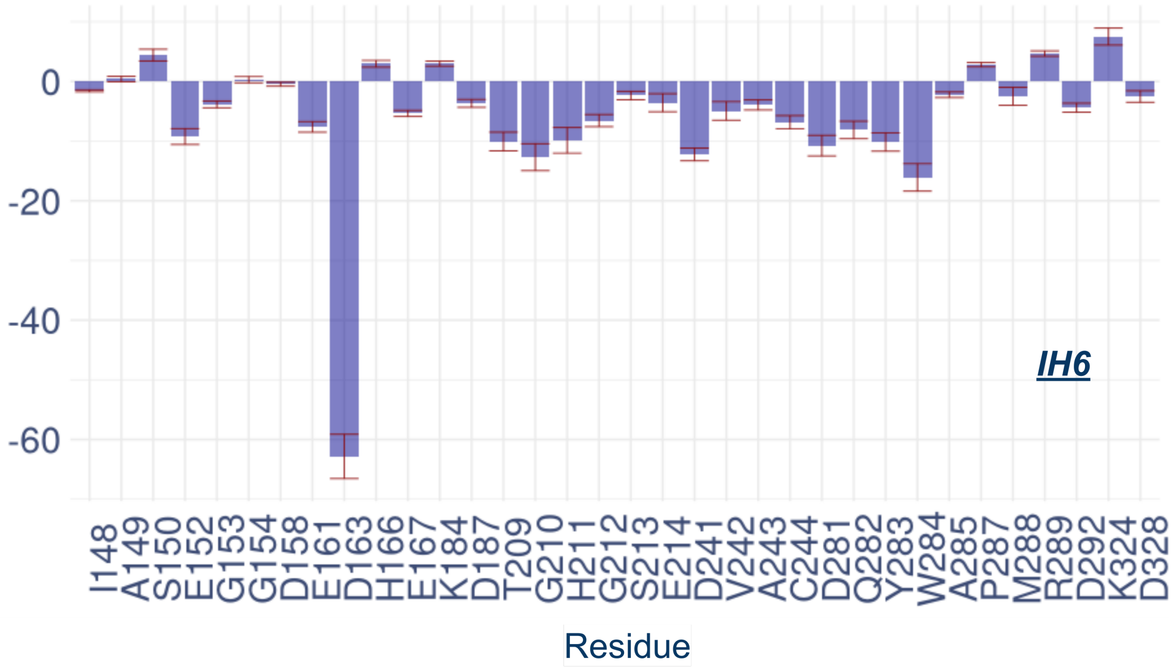

Figure S6. Averaged interaction energies (electrostatic plus Lennard−Jones) between some residues of RgpB and IH6.

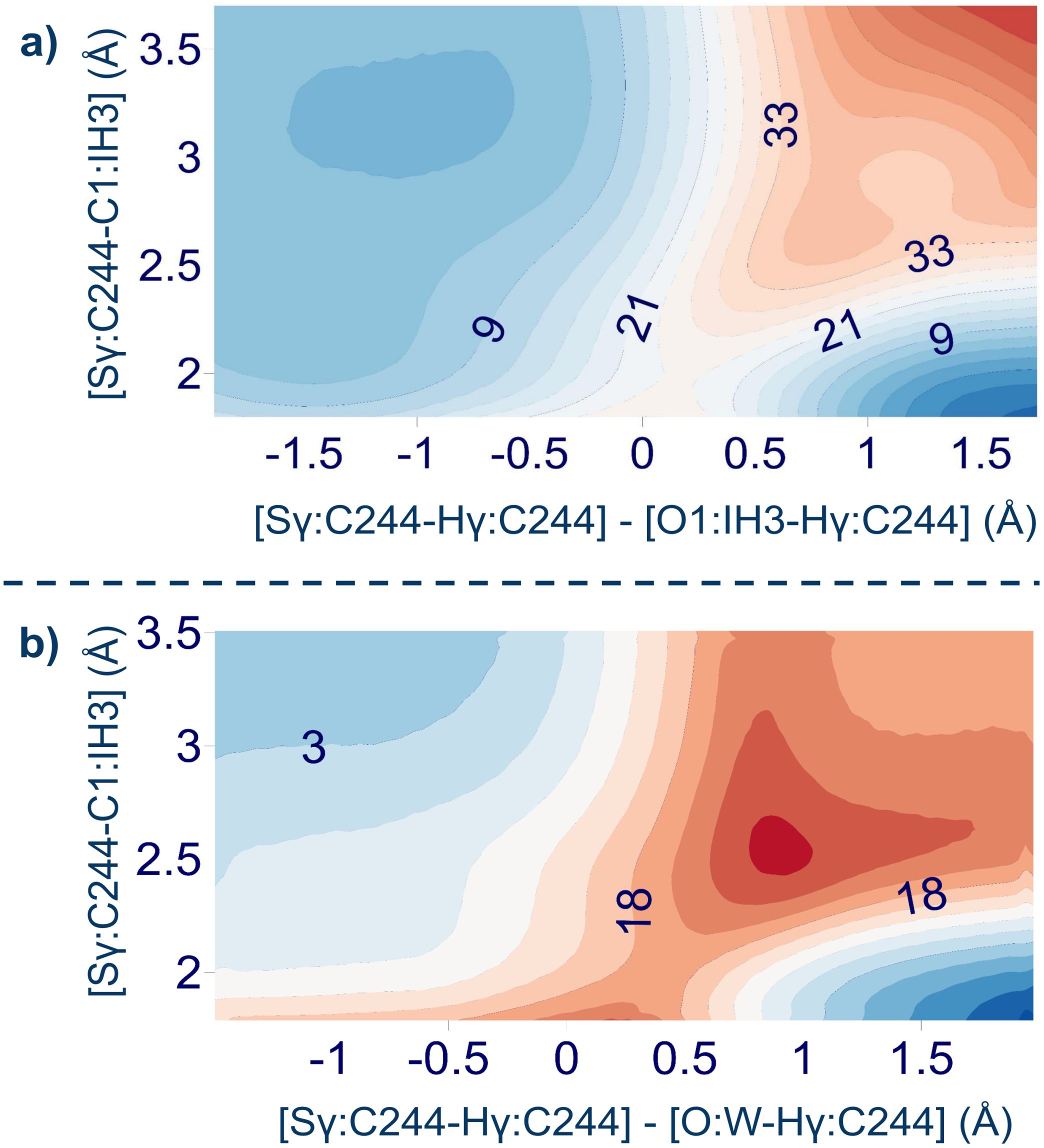

Figure S7. Free energy surfaces computed at PM6/MM level for the reaction mechanisms considered for the covalent binding inhibition of the RgpB gingipain. The values of energies are in kcal·mol^{-1}. Each level in the contour plots represent a 3 kcal·mol^{-1} variation.

IH3 MOL2 FILE:

```
@<TRIPOS>MOLECULE
IH3
   52    53     1     0     0
SMALL
resp


@<TRIPOS>ATOM
      1 N1          1.9400    -1.3680     0.3020 n          1 IH3      -0.726019
      2 H1          1.9450    -1.7450    -0.6390 hn         1 IH3       0.333765
      3 C1          0.7530    -0.6430     0.6970 c3         1 IH3       0.212927
      4 H2          0.2950    -1.1350     1.5670 h1         1 IH3       0.102195
      5 C2          1.0970     0.8330     1.0800 c3         1 IH3      -0.429590
      6 C3         -0.2720    -0.6040    -0.4400 c          1 IH3       0.606860
      7 H3          0.1720     1.3430     1.3620 hc         1 IH3       0.131539
      8 H4          1.7950     0.8190     1.9200 hc         1 IH3       0.131539
      9 C4          1.7490     1.6100    -0.0840 c3         1 IH3       0.096711
     10 O1         -0.0310    -1.1430    -1.5030 o          1 IH3      -0.482681
     11 H5          2.6780     1.1100    -0.3770 hc         1 IH3       0.020502
     12 H6          1.0570     1.6330    -0.9370 hc         1 IH3       0.020502
     13 C5          2.0610     3.0440     0.3520 c3         1 IH3       0.229307
     14 H7          1.1340     3.5520     0.6470 h1         1 IH3       0.038436
     15 H8          2.7520     3.0300     1.2040 h1         1 IH3       0.038436
     16 N2          2.6850     3.7900    -0.7600 n          1 IH3      -0.732111
     17 H9          2.8220     3.2880    -1.6280 hn         1 IH3       0.372142
     18 C6          3.0770     5.0630    -0.7070 c          1 IH3       1.102047
     19 N3          2.9350     5.8020     0.3980 n          1 IH3      -1.038938
     20 N4          3.6300     5.6010    -1.8090 n          1 IH3      -1.038938
     21 H10         2.5220     5.4180     1.2360 hn         1 IH3       0.482854
```

```
22 H11     3.2340   6.7660   0.4310 hn    1 IH3    0.482854
23 H12     3.7480   5.0630  -2.6550 hn    1 IH3    0.482854
24 H13     3.9480   6.5590  -1.8340 hn    1 IH3    0.482854
25 C7      3.0180  -1.5550   1.1290 c     1 IH3    0.894639
26 O2      3.0530  -1.1160   2.2750 o     1 IH3   -0.637783
27 C8      4.1430  -2.3400   0.5090 ca    1 IH3   -0.187791
28 C9      3.9510  -3.6800   0.1600 ca    1 IH3   -0.099678
29 C10     5.3790  -1.7300   0.2810 ca    1 IH3   -0.099678
30 C11     4.9940  -4.4100  -0.4150 ca    1 IH3   -0.125487
31 H14     2.9890  -4.1540   0.3380 ha    1 IH3    0.125902
32 C12     6.4210  -2.4600  -0.2940 ca    1 IH3   -0.125487
33 H15     5.5290  -0.6880   0.5520 ha    1 IH3    0.125902
34 C13     6.2290  -3.8000  -0.6430 ca    1 IH3   -0.099557
35 H16     4.8450  -5.4520  -0.6850 ha    1 IH3    0.138002
36 H17     7.0410  -4.3670  -1.0900 ha    1 IH3    0.137605
37 H18     7.3820  -1.9850  -0.4720 ha    1 IH3    0.138002
38 C14    -1.6110   0.1170  -0.2370 c3    1 IH3   -0.182194
39 H19    -1.4880   0.9900   0.4160 h1    1 IH3    0.114107
40 H20    -2.0140   0.4620  -1.1940 h1    1 IH3    0.114107
41 O3     -2.4820  -0.8470   0.3630 os    1 IH3   -0.280252
42 C15    -3.8240  -0.6280   0.1670 ca    1 IH3    0.114877
43 C16    -4.5610   0.0780   1.1170 ca    1 IH3    0.118702
44 C17    -4.4490  -1.1110  -0.9830 ca    1 IH3    0.118702
45 C18    -5.9230   0.3020   0.9230 ca    1 IH3    0.269084
46 C19    -5.8100  -0.8850  -1.1750 ca    1 IH3    0.269084
47 C20    -6.5470  -0.1810  -0.2260 ca    1 IH3   -0.450119
48 H21    -7.6060  -0.0080  -0.3800 ha    1 IH3    0.278665
49 F1     -3.9580   0.5410   2.2220 f     1 IH3   -0.136356
50 F2     -6.6280   0.9790   1.8360 f     1 IH3   -0.158344
51 F3     -3.7450  -1.7890  -1.8960 f     1 IH3   -0.136356
52 F4     -6.4120  -1.3470  -2.2760 f     1 IH3   -0.158344
@<TRIPOS>BOND
     1     1     2 1
```

2	1	3	1
3	1	25	1
4	3	4	1
5	3	5	1
6	3	6	1
7	5	7	1
8	5	8	1
9	5	9	1
10	6	10	2
11	6	38	1
12	9	11	1
13	9	12	1
14	9	13	1
15	13	14	1
16	13	15	1
17	13	16	1
18	16	17	1
19	16	18	2
20	18	19	1
21	18	20	1
22	19	21	1
23	19	22	1
24	20	23	1
25	20	24	1
26	25	26	2
27	25	27	1
28	27	28	ar
29	27	29	ar
30	28	30	ar
31	28	31	1
32	29	32	ar
33	29	33	1
34	30	34	ar

```
   35    30    35 1
   36    32    34 ar
   37    32    37 1
   38    34    36 1
   39    38    39 1
   40    38    40 1
   41    38    41 1
   42    41    42 1
   43    42    43 ar
   44    42    44 ar
   45    43    45 ar
   46    43    49 1
   47    44    46 ar
   48    44    51 1
   49    45    47 ar
   50    45    50 1
   51    46    47 ar
   52    46    52 1
   53    47    48 1
@<TRIPOS>SUBSTRUCTURE
     1 IH3         1 TEMP              0 ****  ****    0 ROOT
```

IH3 FRCMOD FILE:

```
Remark line goes here
MASS
n  14.010        0.530
hn 1.008         0.161
c3 12.010        0.878
h1 1.008         0.135
c  12.010        0.616
hc 1.008         0.135
o  16.000        0.434
ca 12.010        0.360
ha 1.008         0.135
os 16.000        0.465
f  19.000        0.320

BOND
hn-n   403.20   1.013
c3-n   328.70   1.462
c -n   427.60   1.379
c3-h1  330.60   1.097
c3-c3  300.90   1.538
c -c3  313.00   1.524
c3-hc  330.60   1.097
c -o   637.70   1.218
c -ca  345.90   1.491
ca-ca  461.10   1.398
ca-ha  345.80   1.086
c3-os  308.60   1.432
ca-os  376.60   1.370
ca-f   357.80   1.349
```

ANGLE

h1-c3-n   49.800   108.880

c3-c3-n   65.900   111.610

c -c3-n   67.000   109.060

n -c -o   74.200   123.050

ca-c -n   67.700   115.250

c3-n -hn   45.800   117.680

c -n -hn   48.300   117.550

c -n -c3   63.400   120.690

c3-c3-hc   46.300   109.800

c3-c3-c3   62.900   111.510

c3-c -o   67.400   123.200

c3-c -c3   62.000   116.500

c3-c3-h1   46.400   109.560

c -c3-h1   47.000   108.220

c -c3-c3   63.300   111.040

c -c3-os   68.000   109.210

hc-c3-hc   39.400   107.580

h1-c3-h1   39.200   108.460

n -c -n   72.900   113.560

hn-n -hn   39.600   117.950

c -ca-ca   64.300   120.330

ca-c -o   68.700   122.600

ca-ca-ca   66.600   120.020

ca-ca-ha   48.200   119.880

c3-os-ca   62.500   117.960

h1-c3-os   50.800   109.780

ca-ca-os   69.600   119.200

ca-ca-f   67.100   118.960

DIHE

hc-c3-c3-n   9   1.400   0.000   3.000

c3-c3-c3-n	9	1.400	0.000	3.000
o -c -c3-n	6	0.000	180.000	2.000
c3-c -c3-n	6	0.000	180.000	2.000
n -c -ca-ca	4	4.000	180.000	2.000
h1-c3-n -hn	6	0.000	0.000	2.000
c3-c3-n -hn	6	0.000	0.000	2.000
c -c3-n -hn	6	0.000	0.000	2.000
o -c -n -hn	1	2.500	180.000	-2.000
o -c -n -hn	1	2.000	0.000	1.000
ca-c -n -hn	4	10.000	180.000	2.000
o -c -n -c3	4	10.000	180.000	2.000
ca-c -n -c3	4	10.000	180.000	2.000
c3-c3-c3-hc	1	0.160	0.000	3.000
c3-c3-c3-c3	1	0.180	0.000	-3.000
c3-c3-c3-c3	1	0.250	180.000	-2.000
c3-c3-c3-c3	1	0.200	180.000	1.000
c3-c -c3-h1	6	0.000	180.000	2.000
c3-c -c3-os	6	0.000	180.000	2.000
h1-c3-c3-hc	9	1.400	0.000	3.000
c3-c3-c3-h1	9	1.400	0.000	3.000
o -c -c3-h1	1	0.800	0.000	-1.000
o -c -c3-h1	1	0.000	0.000	-2.000
o -c -c3-h1	1	0.080	180.000	3.000
o -c -c3-c3	6	0.000	180.000	2.000
c3-c -c3-c3	6	0.000	180.000	2.000
c -c3-c3-hc	9	1.400	0.000	3.000
c -c3-c3-c3	9	1.400	0.000	3.000
c -c3-os-ca	3	1.150	0.000	3.000
hc-c3-c3-hc	1	0.150	0.000	3.000
c3-c3-n -c	1	0.500	180.000	-4.000
c3-c3-n -c	1	0.150	180.000	-3.000
c3-c3-n -c	1	0.000	0.000	-2.000
c3-c3-n -c	1	0.530	0.000	1.000

o -c -c3-os 6 0.000 180.000 2.000

n -c -n -c3 4 10.000 180.000 2.000

h1-c3-n -c 6 0.000 0.000 2.000

n -c -n -hn 4 10.000 180.000 2.000

c -c3-n -c 1 0.850 180.000 -2.000

c -c3-n -c 1 0.800 0.000 1.000

c -ca-ca-ca 4 14.500 180.000 2.000

c -ca-ca-ha 4 14.500 180.000 2.000

o -c -ca-ca 4 4.000 180.000 2.000

ca-ca-ca-ca 4 14.500 180.000 2.000

ca-ca-ca-ha 4 14.500 180.000 2.000

ha-ca-ca-ha 4 14.500 180.000 2.000

ca-ca-os-c3 2 1.800 180.000 2.000

h1-c3-os-ca 3 1.150 0.000 3.000

ca-ca-ca-os 4 14.500 180.000 2.000

f -ca-ca-os 4 14.500 180.000 2.000

ca-ca-ca-f 4 14.500 180.000 2.000

f -ca-ca-f 4 14.500 180.000 2.000

f -ca-ca-ha 4 14.500 180.000 2.000

IMPROPER

c -c3-n -hn 1.1 180.0 2.0

c3-c3-c -o 10.5 180.0 2.0 Using general improper torsional angle X- X- c- o, penalty score= 6.0)

n -n -c -n 1.1 180.0 2.0 Using the default value

c -hn-n -hn 1.1 180.0 2.0 Using general improper torsional angle X- X- n-hn, penalty score= 6.0)

ca-n -c -o 10.5 180.0 2.0 Using general improper torsional angle X- X- c- o, penalty score= 6.0)

c -ca-ca-ca 1.1 180.0 2.0 Using the default value

ca-ca-ca-ha 1.1 180.0 2.0 Using general improper torsional angle X- X-ca-ha, penalty score= 6.0)

ca-ca-ca-os 1.1 180.0 2.0 Using the default value

ca-ca-ca-f	1.1	180.0	2.0

NONBON

n	1.8240	0.1700
hn	0.6000	0.0157
c3	1.9080	0.1094
h1	1.3870	0.0157
c	1.9080	0.0860
hc	1.4870	0.0157
o	1.6612	0.2100
ca	1.9080	0.0860
ha	1.4590	0.0150
os	1.6837	0.1700
f	1.7500	0.0610

IHV MOL2 FILE:

```
@<TRIPOS>MOLECULE
IHV
   28    27     1     0     0
SMALL
resp


@<TRIPOS>ATOM
      1 N1           2.8560     0.3100    -0.6880 n          1 IHV      -0.582456
      2 H1           3.0590    -0.3380    -1.4410 hn         1 IHV       0.325682
      3 C1           2.5340    -0.2770     0.5980 c3         1 IHV       0.263671
      4 H2           3.2640     0.0610     1.3490 h1         1 IHV       0.106044
      5 C2           1.1090     0.1440     1.0650 c3         1 IHV      -0.564600
      6 C3           2.5720    -1.7900     0.5320 c          1 IHV       0.320266
      7 H3           0.9090    -0.3310     2.0340 hc         1 IHV       0.162640
      8 H4           1.0850     1.2310     1.1580 hc         1 IHV       0.162640
      9 C4           0.0020    -0.2840     0.0770 c3         1 IHV       0.184094
     10 O1           2.8390    -2.4110    -0.4730 o          1 IHV      -0.436977
     11 H5           0.1860     0.1820    -0.8970 hc         1 IHV       0.026178
     12 H6           0.0120    -1.3760    -0.0290 hc         1 IHV       0.026178
     13 C5          -1.3650     0.1620     0.6010 c3         1 IHV       0.187846
     14 H7          -1.5580    -0.3010     1.5770 h1         1 IHV       0.047366
     15 H8          -1.3840     1.2530     0.7090 h1         1 IHV       0.047366
     16 N2          -2.4280    -0.2450    -0.3410 n          1 IHV      -0.736190
     17 H9          -2.1390    -0.7420    -1.1730 hn         1 IHV       0.355037
     18 C6          -3.7310    -0.0070    -0.1770 c          1 IHV       1.153561
     19 N3          -4.2040     0.6420     0.8910 n          1 IHV      -1.089551
     20 N4          -4.5770    -0.4430    -1.1280 n          1 IHV      -1.089551
     21 H10         -3.5860     0.9780     1.6160 hn         1 IHV       0.500425
```

22 H11 -5.1920 0.8180 1.0070 hn 1 IHV 0.500425

23 H12 -4.2440 -0.9360 -1.9440 hn 1 IHV 0.500425

24 H13 -5.5740 -0.2960 -1.0640 hn 1 IHV 0.500425

25 C7 2.8950 1.6540 -0.9090 c 1 IHV 0.568545

26 O2 2.6660 2.5030 -0.0630 o 1 IHV -0.562933

27 H14 2.3340 -2.3010 1.4870 h4 1 IHV 0.076394

28 H15 3.1600 1.9000 -1.9540 h5 1 IHV 0.047051

@<TRIPOS>BOND

1 1 2 1

2 1 3 1

3 1 25 1

4 3 4 1

5 3 5 1

6 3 6 1

7 5 7 1

8 5 8 1

9 5 9 1

10 6 10 2

11 6 27 1

12 9 11 1

13 9 12 1

14 9 13 1

15 13 14 1

16 13 15 1

17 13 16 1

18 16 17 1

19 16 18 2

20 18 19 1

21 18 20 1

22 19 21 1

23 19 22 1

24 20 23 1

25 20 24 1

```
   26    25    26 2
   27    25    28 1
@<TRIPOS>SUBSTRUCTURE
     1 IHV         1 TEMP              0 ****  ****    0 ROOT
```

IHV FRCMOD FILE:

Remark line goes here

MASS

n 14.010   0.530

hn 1.008   0.161

c3 12.010   0.878

h1 1.008   0.135

c 12.010   0.616

hc 1.008   0.135

o 16.000   0.434

nh 14.010   0.530

cz 12.010   0.360

h4 1.008   0.135

h5 1.008   0.135

BOND

hn-n   403.20   1.013

c3-n   328.70   1.462

c -n   427.60   1.379

c3-h1 330.60   1.097

c3-c3 300.90   1.538

c -c3 313.00   1.524

c3-hc 330.60   1.097

c -o   637.70   1.218

c -h4 310.70   1.112

c3-nh 326.60   1.464

hn-nh 404.60   1.012

cz-nh 488.00   1.339

c -h5 319.70   1.105

ANGLE

h1-c3-n 49.800 108.880

c3-c3-n 65.900 111.610

c -c3-n 67.000 109.060

n -c -o 74.200 123.050

h5-c -n 51.400 112.160

c3-n -hn 45.800 117.680

c -n -hn 48.300 117.550

c -n -c3 63.400 120.690

c3-c3-hc 46.300 109.800

c3-c3-c3 62.900 111.510

c3-c -o 67.400 123.200

c3-c -h4 45.600 114.640

c3-c3-h1 46.400 109.560

c -c3-h1 47.000 108.220

c -c3-c3 63.300 111.040

hc-c3-hc 39.400 107.580

c3-c3-nh 66.200 110.460

h4-c -o 54.200 120.700

c3-nh-hn 46.100 115.990

c3-nh-cz 62.900 125.460

h1-c3-h1 39.200 108.460

h1-c3-nh 49.600 109.790

nh-cz-nh 73.000 120.140

cz-nh-hn 48.800 121.150

hn-nh-hn 40.100 115.120

h5-c -o 53.700 123.650

DIHE

hc-c3-c3-n 9 1.400 0.000 3.000

c3-c3-c3-n 9 1.400 0.000 3.000

o -c -c3-n 6 0.000 180.000 2.000

h4-c -c3-n 6 0.000 180.000 2.000

h1-c3-n -hn	6	0.000	0.000	2.000	
c3-c3-n -hn	6	0.000	0.000	2.000	
c -c3-n -hn	6	0.000	0.000	2.000	
o -c -n -hn	1	2.500	180.000	-2.000	
o -c -n -hn	1	2.000	0.000	1.000	
h5-c -n -hn	4	10.000	180.000	2.000	
o -c -n -c3	4	10.000	180.000	2.000	
h5-c -n -c3	4	10.000	180.000	2.000	
c3-c3-c3-hc	1	0.160	0.000	3.000	
c3-c3-c3-c3	1	0.180	0.000	-3.000	
c3-c3-c3-c3	1	0.250	180.000	-2.000	
c3-c3-c3-c3	1	0.200	180.000	1.000	
h1-c3-c3-hc	9	1.400	0.000	3.000	
c3-c3-c3-h1	9	1.400	0.000	3.000	
o -c -c3-h1	1	0.800	0.000	-1.000	
o -c -c3-h1	1	0.000	0.000	-2.000	
o -c -c3-h1	1	0.080	180.000	3.000	
h4-c -c3-h1	6	0.000	180.000	2.000	
o -c -c3-c3	6	0.000	180.000	2.000	
h4-c -c3-c3	6	0.000	180.000	2.000	
c3-c3-c3-nh	9	1.400	0.000	3.000	
c -c3-c3-hc	9	1.400	0.000	3.000	
c -c3-c3-c3	9	1.400	0.000	3.000	
hc-c3-c3-hc	1	0.150	0.000	3.000	
c3-c3-nh-hn	6	0.000	0.000	2.000	
c3-c3-nh-cz	6	0.000	0.000	2.000	
hc-c3-c3-nh	9	1.400	0.000	3.000	
nh-cz-nh-c3	4	2.700	180.000	2.000	same as X -c2-nh-X , penalty score=462.5
h1-c3-nh-hn	6	0.000	0.000	2.000	
h1-c3-nh-cz	6	0.000	0.000	2.000	
nh-cz-nh-hn	4	2.700	180.000	2.000	same as X -c2-nh-X , penalty score=462.5
h1-c3-n -c	6	0.000	0.000	2.000	
c3-c3-n -c	1	0.500	180.000	-4.000	

c3-c3-n -c 1 0.150 180.000 -3.000

c3-c3-n -c 1 0.000 0.000 -2.000

c3-c3-n -c 1 0.530 0.000 1.000

c -c3-n -c 1 0.850 180.000 -2.000

c -c3-n -c 1 0.800 0.000 1.000

IMPROPER

c -c3-n -hn 1.1 180.0 2.0

c3-h4-c -o 10.5 180.0 2.0 Using general improper torsional angle X- X- c- o, penalty score= 6.0)

c3-cz-nh-hn 1.1 180.0 2.0 Using the default value

nh-nh-cz-nh 1.1 180.0 2.0 Using the default value

cz-hn-nh-hn 1.1 180.0 2.0 Same as X -X -na-hn, penalty score= 41.2 (use general term))

h5-n -c -o 10.5 180.0 2.0 Using general improper torsional angle X- X- c- o, penalty score= 6.0)

NONBON

n 1.8240 0.1700

hn 0.6000 0.0157

c3 1.9080 0.1094

h1 1.3870 0.0157

c 1.9080 0.0860

hc 1.4870 0.0157

o 1.6612 0.2100

nh 1.8240 0.1700

cz 1.9080 0.0860

h4 1.4090 0.0150

h5 1.3590 0.0150

QM REGION COORDINATES FOR TRANSITION STATE (TS_W) STRUCTURE:

96

C	47.624249	81.588348	62.890717
H	47.985661	80.606384	62.530949
H	47.235641	82.132645	62.014744
C	48.876663	82.286835	63.496735
O	49.399414	83.201538	62.739300
O	49.314838	81.875092	64.590317
C	52.514141	84.329651	57.975819
O	52.967987	85.315399	58.591171
N	53.189388	83.172523	57.843319
H	52.873268	82.488045	57.151871
C	54.491104	82.899460	58.443508
H	54.989918	83.868607	58.619629
C	54.297817	82.200020	59.826122
H	53.805382	81.227097	59.650463
H	55.299221	81.993080	60.239849
C	53.492695	82.987503	60.825127
N	53.986176	84.101418	61.496586
C	52.992359	84.463181	62.329380
H	53.065025	85.285957	63.041138
N	51.894096	83.658714	62.229549
H	50.889011	83.583122	62.714272
C	52.205009	82.722893	61.274761
H	51.488773	81.939369	61.045029
C	55.292084	82.085571	57.379807
O	54.838585	81.932480	56.241741
N	56.488247	81.529961	57.772121
H	56.864220	81.890640	58.652100

C	57.319881	80.719765	56.840694
H	56.693230	80.574890	55.948055
H	58.205940	81.297165	56.519791
C	58.588017	85.155396	55.607357
O	59.435051	85.732475	54.920357
N	58.942921	84.408653	56.717377
H	58.197227	83.967979	57.265423
C	60.357407	84.137390	57.129753
H	60.861404	85.097099	57.326214
C	60.292995	83.269890	58.418858
H	59.416660	82.592407	58.334961
H	61.180019	82.617241	58.468620
S	60.252052	84.013885	60.125420
H	59.858013	82.354027	60.579681
N	58.031010	86.013763	61.984367
H	59.043861	85.911034	61.941349
C	57.292057	85.265121	60.975861
H	56.249912	85.136116	61.362591
C	57.243580	86.039726	59.650162
C	57.836182	83.837532	60.972218
H	58.277637	86.245583	59.323738
H	56.779430	85.413284	58.865749
C	56.456009	87.359116	59.819996
O	57.412155	82.955727	60.106564
H	56.712597	87.810471	60.794395
H	56.756618	88.077858	59.035427
C	54.952545	87.092468	59.741367
H	54.689339	86.712021	58.740952
H	54.648239	86.315094	60.466152
N	54.154266	88.298431	60.026821
H	54.546959	89.205368	59.773037
C	52.866600	88.282593	60.410492
N	52.138428	87.168312	60.364517

N	52.299870	89.462830	60.815521
H	51.319454	87.057076	60.959938
H	52.414272	86.360451	59.753647
H	52.864910	90.090050	61.386295
H	51.309010	89.449898	61.048088
C	57.461437	86.522888	63.129932
O	56.232346	86.653954	63.245193
C	58.413204	86.914581	64.236282
C	59.809631	86.719696	64.203552
C	57.838734	87.461891	65.398819
C	60.607372	87.088608	65.292809
H	60.304302	86.239594	63.353630
C	58.634380	87.837006	66.485268
H	56.752354	87.552536	65.433685
C	60.024380	87.656265	66.433800
H	61.684834	86.901726	65.255653
H	60.650082	87.942924	67.284973
H	58.167557	88.259155	67.380859
C	58.002571	83.278465	62.400146
H	57.095287	83.608543	62.935635
H	58.013069	82.177238	62.386284
O	59.192764	83.753822	63.049622
C	59.350502	83.567299	64.393921
C	58.357269	83.305252	65.359756
C	60.671997	83.749725	64.856606
C	58.699554	83.222214	66.719734
C	60.986938	83.680016	66.213646
C	60.006706	83.402115	67.167931
H	60.249889	83.341789	68.229630
F	57.054165	83.147308	65.027184
F	57.713142	82.967545	67.599609
F	61.627850	84.079956	63.965172
F	62.264027	83.899521	66.587868

O	59.205906	81.383141	60.611393
H	59.493832	80.827522	59.856094
H	58.213539	81.966354	60.326187

QM REGION COORDINATES FOR REACTANTS (R) STRUCTURE:

96

C	47.619030	81.652100	62.917931
H	47.930462	80.691505	62.486317
H	47.238106	82.266220	62.101234
C	48.854809	82.261345	63.563835
O	49.458378	83.169693	62.877743
O	49.251507	81.852386	64.652275
C	52.554493	84.313766	57.990585
O	53.134624	85.319061	58.422516
N	53.172291	83.085320	58.033230
H	52.744503	82.256271	57.617256
C	54.531349	82.902992	58.591866
H	55.006588	83.911484	58.775005
C	54.425426	82.154243	59.938068
H	54.000816	81.139549	59.783466
H	55.444881	82.000282	60.351742
C	53.595566	82.894234	60.933346
N	54.031582	84.024338	61.627106
C	53.003342	84.335754	62.490551
H	53.038746	85.136429	63.210556
N	51.934547	83.459236	62.358765
H	50.885906	83.393555	62.900272
C	52.300247	82.556084	61.372246
H	51.655319	81.753777	61.080029
C	55.341171	82.159569	57.505238

O	54.905567	82.002022	56.376041
N	56.593189	81.650124	57.825058
H	57.067860	81.916367	58.689701
C	57.325733	80.800293	56.840263
H	56.681171	80.628952	55.950943
H	58.232609	81.317307	56.486176
C	58.580002	85.116982	55.811672
O	59.478336	85.650558	55.170868
N	58.938339	84.376205	56.953995
H	58.250309	84.006187	57.609718
C	60.394135	84.142677	57.262081
H	60.889664	85.135872	57.374752
C	60.564884	83.283676	58.518623
H	60.039143	82.309006	58.437901
H	61.641384	83.066490	58.680569
S	59.977577	84.121994	60.026093
H	60.106544	83.089928	60.911839
N	58.152016	85.928185	62.274651
H	59.118172	85.600319	62.247219
C	57.265636	85.491150	61.181919
H	56.177200	85.644661	61.517956
C	57.510731	86.287369	59.885815
C	57.393673	83.981606	60.984425
H	58.567341	86.624252	59.826946
H	57.374828	85.629143	59.002033
C	56.576904	87.499908	59.812447
O	57.185608	83.436493	59.917519
H	56.563446	88.005440	60.806480
H	56.979748	88.245552	59.102554
C	55.163853	87.055634	59.413750
H	55.089539	86.779892	58.333702
H	54.869896	86.128983	59.976357
N	54.204926	88.163948	59.690037

H	54.496338	89.100258	59.409351
C	52.957371	87.968269	60.226891
N	52.491096	86.738342	60.585888
N	52.138123	89.099785	60.403099
H	51.547501	86.598198	60.932854
H	52.974819	85.869293	60.260418
H	52.578228	90.005951	60.523102
H	51.288803	88.996475	60.952667
C	57.665821	86.743706	63.300457
O	56.516346	87.160828	63.234066
C	58.598293	87.050323	64.428070
C	59.984734	86.841019	64.372543
C	58.026306	87.590256	65.591118
C	60.787197	87.171761	65.466377
H	60.454578	86.431007	63.477837
C	58.832932	87.932838	66.678398
H	56.945030	87.734337	65.640907
C	60.213360	87.723663	66.616966
H	61.862041	87.001968	65.423752
H	60.840851	87.993660	67.465263
H	58.383060	88.360291	67.574249
C	57.543045	83.127609	62.251957
H	56.686733	83.358826	62.916035
H	57.577499	82.044701	62.029530
O	58.845268	83.441170	62.825714
C	59.025730	83.402893	64.181908
C	58.110134	83.183212	65.224686
C	60.380009	83.638023	64.539268
C	58.544132	83.227760	66.564110
C	60.784962	83.653351	65.875420
C	59.872490	83.455170	66.918098
H	60.189770	83.475769	67.963387
F	56.826027	82.920624	64.998749

F	57.650631	83.036926	67.525543
F	61.257942	83.826042	63.559460
F	62.062740	83.862968	66.168648
O	59.020229	81.447556	60.360493
H	59.034721	80.501060	60.484417
H	58.182602	81.675430	59.930988

QM REGION COORDINATES FOR PRODUCT (P) STRUCTURE:

96

C	47.618038	81.639740	62.923149
H	47.946659	80.676514	62.511894
H	47.238941	82.239136	62.094959
C	48.827061	82.274765	63.587093
O	49.391190	83.219872	62.919884
O	49.242867	81.855927	64.665451
C	52.527370	84.318550	58.035431
O	53.130333	85.326347	58.419315
N	53.084499	83.068665	58.198254
H	52.606453	82.227005	57.879925
C	54.445545	82.882973	58.741196
H	54.930874	83.895096	58.897675
C	54.373547	82.167603	60.105595
H	53.981419	81.136208	59.986385
H	55.399391	82.070061	60.522785
C	53.528046	82.910553	61.086201
N	53.972340	84.031166	61.789093
C	52.946278	84.349831	62.648632
H	52.987690	85.142563	63.376839
N	51.866165	83.489258	62.499241
H	50.814831	83.447929	63.018196

C	52.223640	82.587090	61.509007
H	51.565517	81.801254	61.199379
C	55.230415	82.155380	57.626453
O	54.886082	82.187569	56.457985
N	56.394520	81.472015	57.960712
H	56.703934	81.399399	58.929409
C	57.175476	80.753708	56.907406
H	56.530792	80.578033	56.022045
H	58.021664	81.375763	56.571800
C	58.577030	85.134399	55.785793
O	59.479023	85.644379	55.133453
N	58.912357	84.405533	56.937733
H	58.200169	84.110146	57.613831
C	60.352734	84.138344	57.287708
H	60.875732	85.113022	57.431206
C	60.353416	83.308266	58.589130
H	59.685753	82.425400	58.515045
H	61.379120	82.929153	58.788349
S	59.997543	84.195732	60.162758
H	59.607273	81.454323	60.873539
N	58.334320	85.974419	62.145943
H	59.341972	85.798340	62.187492
C	57.627186	85.487236	60.939724
H	56.523182	85.351830	61.205536
C	57.719635	86.448700	59.745930
C	58.206066	84.073830	60.651325
H	58.737560	86.879906	59.663620
H	57.566132	85.883324	58.803284
C	56.684608	87.574867	59.869183
O	57.502934	83.520950	59.566433
H	56.814228	88.081329	60.853638
H	56.874718	88.341789	59.096359
C	55.262875	87.006477	59.763504

H	55.074162	86.509033	58.778816
H	55.119400	86.221931	60.562210
N	54.272537	88.106606	59.951012
H	54.568531	89.045822	59.686600
C	52.976685	87.882858	60.339718
N	52.504604	86.642426	60.651810
N	52.115894	88.996841	60.421253
H	51.538139	86.483124	60.917274
H	53.025913	85.786026	60.366444
H	52.521786	89.908035	60.607151
H	51.214500	88.869377	60.874767
C	57.702698	86.685707	63.173000
O	56.531475	87.018417	63.063381
C	58.553169	87.008026	64.366692
C	59.950386	86.868668	64.386467
C	57.897926	87.494148	65.508347
C	60.679604	87.234619	65.519142
H	60.486839	86.479614	63.519054
C	58.629639	87.870522	66.637970
H	56.809498	87.566032	65.509605
C	60.021416	87.746246	66.643257
H	61.763523	87.124748	65.526749
H	60.593479	88.045135	67.521400
H	58.110828	88.257912	67.514549
C	57.993183	83.220192	61.923492
H	57.114536	83.599632	62.485889
H	57.823799	82.143776	61.726776
O	59.233646	83.323936	62.679924
C	59.191051	83.377937	64.047760
C	58.132179	83.153931	64.946121
C	60.464268	83.672989	64.602501
C	58.346203	83.276321	66.333206
C	60.654205	83.753662	65.983322

C	59.594818	83.569656	66.879105
H	59.741795	83.649231	67.958458
F	56.915462	82.806969	64.534943
F	57.320648	83.091530	67.154282
F	61.478100	83.852341	63.762718
F	61.865356	84.016724	66.460701
O	58.822933	81.131874	60.400723
H	58.874947	80.177597	60.390125
H	57.759945	82.549774	59.460674
